# A prospective analysis of mucosal microbiome-metabonome interactions in colorectal cancer using a combined MAS 1HNMR and metataxonomic strategy

**DOI:** 10.1038/s41598-017-08150-3

**Published:** 2017-08-21

**Authors:** James Kinross, Reza Mirnezami, James Alexander, Richard Brown, Alasdair Scott, Dieter Galea, Kirill Veselkov, Rob Goldin, Ara Darzi, Jeremy Nicholson, Julian R. Marchesi

**Affiliations:** 1 Division of Surgery, Department of Surgery and Cancer, Imperial College London, London, UK; 2 Computational and Systems Medicine, Department of Surgery and Cancer, Faculty of Medicine, Imperial College London, London, UK; 3 Centre for Pathology, Faculty of Medicine, Imperial College London, London, UK; 4 Division of Digestive Diseases, Faculty of Medicine, Department of Surgery and Cancer, Imperial College London, London, UK; 5 School of Biosciences, Cardiff University, Cardiff, UK

## Abstract

Colon cancer induces a state of mucosal dysbiosis with associated niche specific changes in the gut microbiota. However, the key metabolic functions of these bacteria remain unclear. We performed a prospective observational study in patients undergoing elective surgery for colon cancer without mechanical bowel preparation (n = 18). Using 16 S rRNA gene sequencing we demonstrated that microbiota ecology appears to be cancer stage-specific and strongly associated with histological features of poor prognosis. Fusobacteria (p < 0.007) and ε- *Proteobacteria* (p < 0.01) were enriched on tumour when compared to adjacent normal mucosal tissue, and fusobacteria and β-*Proteobacteria* levels increased with advancing cancer stage (p = 0.014 and 0.002 respecitvely). Metabonomic analysis using 1H Magic Angle Spinning Nuclear Magnetic Resonsance  (MAS-NMR) spectroscopy, demonstrated increased abundance of taurine, isoglutamine, choline, lactate, phenylalanine and tyrosine and decreased levels of lipids and triglycerides in tumour relative to adjacent healthy tissue. Network analysis revealed that bacteria associated with poor prognostic features were not responsible for the modification of the cancer mucosal metabonome. Thus the colon cancer mucosal microbiome evolves with cancer stage to meet the demands of cancer metabolism. Passenger microbiota may play a role in the maintenance of cancer mucosal metabolic homeostasis but these metabolic functions may not be stage specific.

## Introduction

Sporadic colorectal cancer (CRC) is the third commonest cause of cancer-related death worldwide and its global incidence is increasing^1^. There is strong epidemiological evidence to suggest that diet (high in red meat and fat; low in fiber) is a risk factor that may explain this trend^2^, but the interaction between the colon and its environment is complex and subject to personalized variation and dynamic xeno-metabolite interactions. Nevertheless, data now exist to support the hypothesis that an important environmental driver of CRC risk is the colonic microbiota and its associated metabonome^3, 4^. For example, it has been demonstrated that the metabolic function of the colonic microbiota directly influences cancer risk through its modulation of dietary fiber, an increase of which leads to profound changes in colonic ecological co-occurrence networks with resulting upregulation of butyrate metabolism and a reduction in the metabolism of secondary bile acids^3^.

Several competing theories regarding the microbial regulation of CRC have now emerged to explain the function and importance of the CRC-associated metagenome (the catalogue of microbial genes that reside within the gut). The keystone-pathogen hypothesis^5^ and the Alpha-Bug hypothesis state that certain low abundance microbiome members may possess unique virulence or amensalistic traits, or produce carcinogens, which are not only pro-oncogenic, but also promote a mucosal immune response and colonic epithelial cell changes that initiate colorectal carcinogenesis^6^. Tjalsma and colleagues proposed the alternative ‘driver passenger’ model for CRC, whereby a ‘first hit’ by indigenous intestinal ‘driver’ bacteria causes epithelial DNA damage, which in turn contributes to the initiation of CRC^7^. The consequent developing tumor induces intestinal niche alterations that favour the proliferation of opportunistic bacteria (termed bacterial ‘passengers’). Pathobionts are commensal organisms that can cause disease when specific genetic or environmental conditions are altered in the host. Colonic pathobionts may be able to influence host pathogenesis through a large number of chemical and molecular signaling pathways. Whether these pathobionts create a specific mucosal metabolic microenvironment that potentiates tumour growth remains unclear.

Important weaknesses of many existing studies examining the colonic microbiota in cancer aetiology are the employment of heterogeneous sampling approaches, the limited oncological phenotyping data presented and the variable use of mechanical bowel preparation^8–16^. This heterogeneity is critical as such variation is likely to influence the ecological characteristics of the microbiota: for example recent data from patients undergoing lower gastrointestinal tract endoscopy have identified short-term changes in the colonic microbiome caused by use of mechanical bowel preparation^17, 18^.

The metabolism of colon cancer is complex, and although fundamental changes in faecal amino acid and microbial co-metabolites (such as choline) have been demonstrated, mucosal microbiome-host metabolic interactions have yet to be fully defined^19–21^. The primary aim of this study, therefore, was to describe the variation in local colonic dysbiosis between tumor and normal mucosa in a homogeneous group of CRC patients, in the absence of mechanical bowel preparation. Further, we aimed to determine the local ecology of the cancer microbiome in the context of cancer phenotype and stage, as defined by the ‘passenger-driver hypothesis’. The secondary aim was to describe mucosal microbiome-metabonome interactions that modulate metabolism at the level of the CRC mucosa^20, 21^.

## Results

### Patient Demographics

Demographic and clinicopathological data for the 18 patients included in the study are summarized in Table 1. There were no intraoperative complications and all patients made a routine recovery. The majority of cases were stage T3 and T4 tumours. Three patients had large tubulovillous adenomas with low grade dysplasia, which were resected due to concerning features suggestive of cancer on pre-operative imaging. A single T1 lesion (pT1N1, Dukes C1) and a T2 lesion were also analysed (pT2N0, Dukes B). Seven of 18 patients had nodal micrometastases (N1/N2). No patients had evidence of distant metastatic disease at the time of surgery. The majority of lesions were adenocarcinomas (15 out of 18) of which five were of mucinous type. Five patients had extramural vascular invasion (EMVI), which is a histological feature of poor prognosis, and two of these were found to express the KRAS mutation.

**Table 1:** Patient demographic and pathological data.

Age (Median)	76 (55–85)
Sex (M:F)	10:8
BMI (Mean)	26.6 (21–39)
Anatomical location	
Ascending colon	16
Descending colon	2
**T stage**	
T0 (TVAs)	3
T1	1
T2	1
T3	5
T4	8
**N stage**	
N0	11
N1	4
N2	3
**Histological subtype**	
Adenocarcinoma	15
Dysplasia	3
Differentiation	
Well	1
Moderate	11
Poor	6
Perineural invasion	1
Lymphovascular invasion (LVI)	6
Extramural vascular invasion (EMVI)	5
KRAS mutants	2

BMI: Body Mass Index; TVA: Tubulovillous adenoma; KRAS: Kirsten rat sarcoma viral oncogene homolog.

### Metataxonomy data

**Figure 1:**
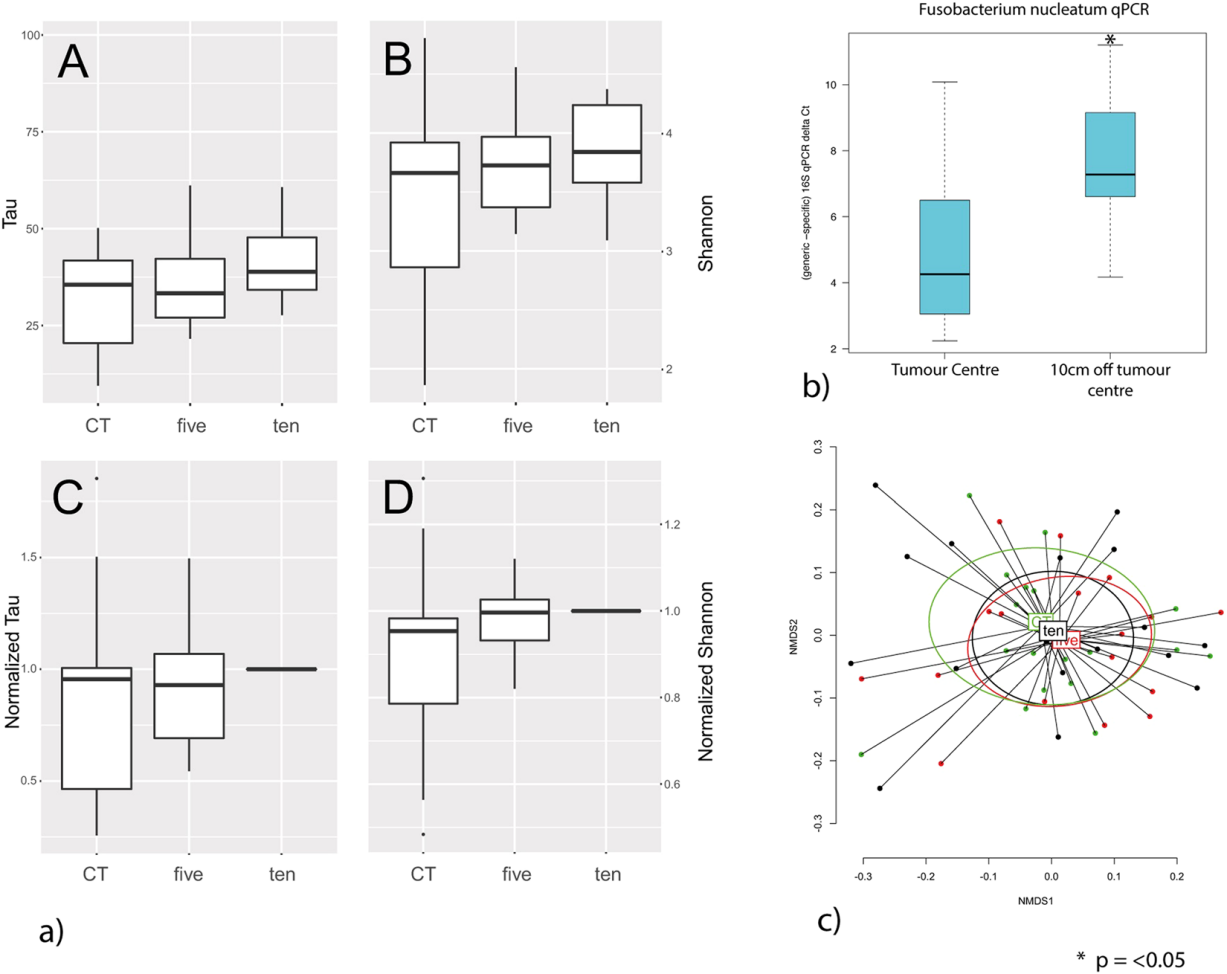
**(a)** Boxplots of the ecological Indices for the tumour (CT, n = 18), 5 cm off the tumour (five, n = 18) and 10 cm off the tumour (ten, n = 18). Each boxplot was calculated from the individual Tau index (A), Shannon index (B), Normalized Tau (values normalized to the on tumour (CT) value for each individual) (C) and Normalized Shannon (values normalized to the on tumour (CT) value for each individual) (D). No statistical differences were observed between the values. (**b**). Box plot of *Fusobacterium nucleatum* 16 S qPCR data, demonstrating over-representation of *Fusobacterium nucleatum* on cancer, when compared to mucosa biopsied at 10cms (p < 0.05). (**c**). PCA plot of non-metric multi-dimensional scaling of OTU data from 18 patients in this study, confirming that there was no multivariate statistically significant ecological variance between on tumour, 5 cm off tumour and 10 cm off tumour sampling points.

There were no significant differences in Tau or Shannon indices of diversity when comparing samples on tumour, 5 cm off tumour and 10 cm off tumour (Fig. 1a), although there was a trend observed towards increased diversity off tumour, which was greatest at 10 cm. This finding was confirmed by non-metric multidimensional scaling of Operational Taxanomic Unit (OTU) data (Fig. 1c) which showed no significant class separation according to tumour, 5 cm and 10 cm sites. However, as previously described in other studies, the 16 s rRNA data demonstrated that *Fusobacterium* was over-represented in cancer biopsies. 8/18 (44%) patients had *Fusobacterium* both on and off tumour. 6/18 (33%) patients did not have *Fusobacterium* on the tumour, and of these only one patient had *Fusobacterium* at an off cancer site (at 5 cm). 4/18 patients (22%) only had *Fusobacterium* on tumour. In keeping with these finidngs, *Fusobacterium nucleatum-*specific qPCR analysis confirmed over-representation of this species on tumour compared to 10 cm off tumour (p < 0.05) (Fig. 1b).

**Figure 2:**
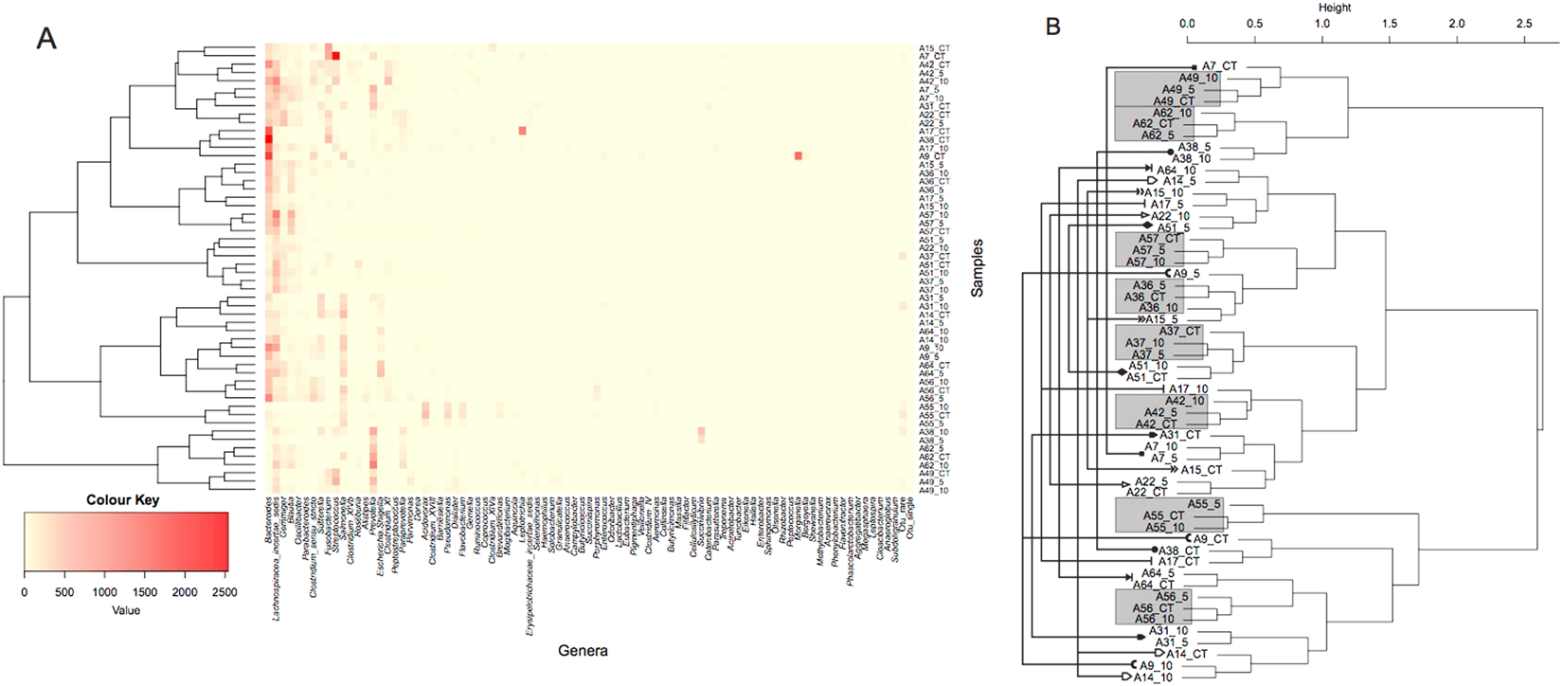
(**A**) Heatmap and (**B**) Clustering dendrogram (Bray-Curtis and Ward linkage) for genus-level OTU data. Figure A. demonstrates that three clusters of mucosal bacteria predmoninate and that on and off cancer samples appear to cluster together. In (**B**) the grey boxes highlight samples taken from tumour, 5 cm off tumour and 10 cm off tumour from the same individuals which cluster together. This shows that 8 out of 18 patients demonstrated significant homogeneity in mucosal bacteria on cancer and at 5 and 10 cms. This clustering effect is greater than any clustering associated with sampling location, suggesting that individual variation is a greater influence on CRC microbiome dysbiosis than colonic geographical sampling site relative to the tumour.

Dendrogram analysis (Fig. 2A) demonstrated three dominant bacterial clusters: 1) *Bacteroide*s, *Lachnospiracea intertie sedis*, *Blautia, Fusobacterium and Streptococcus;* 2) *Bacteroide*s, *Lachnospiracea intertie sedis, Clostridium sensu strictu, Sutterella, Salmonella*, and Escherichia/Shigella; 3) *Lachnospiracea intertie sedis Streptococcus*, *Prevotella* and *Paraprevotella*. Using multivariate statistical modelling, these clusters could be categorized by T stage (p = 0.04), histological subtype (p = 0.05) and tumour differentiation (p = 0.011) (Supplementary data, Table 1). Univariate statistical analysis demonstrated Cluster 1 and 2 contained patients with dysplastic lesions and less advanced cancers, and class 2 only contained patients with moderately differentiated tumours. Cluster 3 (8 patients) was made up of patients with T4 adenocarcinomas with poor tumour differentiation, and a trend towards nodal metastases.

On and off tumour samples from individual patients also clustered together (Fig. 2B). 8 out of 18 patients had on tumour, 5 cm and 10 cm samples that clustered together. These patients tended to have histological features associated with a better oncological prognosis. The absence of nodal metastases (p = 0.017) and moderate tumour differentiation (p = 0.038) were statistically significant for factors for differentiating between these two cohorts, with non-significant trends also noted for T stage, histological subtype and Lymphovascular invasion (LVI) (Supplementary data Table 2). Collectively, these data suggest that individual patient variation has a greater influence on CRC microbiome dysbiosis than colonic geographical sampling site relative to the tumour. Perhaps more critically, the mucosal tumour phenotype is associated with three distinct microbial structures, and ecological heterogeneity between on and off tumour biopsy sites from the same patient is associated with poorer prognostic features on histological examination.

These findings were further investigated using a univariate analysis of the entire 16 S rRNA data set from tumour, 5 cm and 10 cm sites. (Table 2). Histologically poorly differentiated tumors had higher relative abundance of *Fusobacterium* (P < 0.03). Three classes of bacteria belonging to the *Firmicutes* (*F. streptococcus* spp. ( < 0.03), *F. Solobacterium* spp. (P < 0.01). and *Clostridium XI* spp. (P < 0.04)) were also over represented in poorly differentiated tumours while *F*. *subdoligranulum* spp. was under represented (P < 0.01). When a univariate analysis was applied to other histological biomarkers of poor prognosis, such as extra-mural vascular invasion (EMVI), LVI and KRAS mutation status, there were no statistically significant associations with abundance of *Fusobacterium* (data not shown). However, increased *Bacteroidetes*, *Bacteroides* spp. abundance was associated with EMVI (p < 0.03) and *Firmicutes*, *Roseburia* spp. was associated with the presence of LVI (p < 0.02). *Proteobacteria, Aggregatibacter* spp. (P < 0.01) was associated with KRAS mutation (P < 0.01), although this finding requires caution as the patient numbers expressing KRAS mutation were small.

**Table 2:** Summary data of bacteria statistically over- or under-expressed in tumour samples and their statistical association with established histological biomarkers of poor prognosis.

EMVI	P	KRAS	p	LVI	p	Differentiation	p
Bacteroidetes Bacteroides **↑**	0.03	Proteobacteria Aggregatibacter ↑	0.01	Firmicutes Roseburia ↓	0.02	Firmicutes Streptococcus ↑	0.029
						Firmicutes Solobacterium ↑	0.01
						Firmicutes Clostridium XI ↑	0.039
						Firmicutes Subdoligranulum ↓	0.01
						Fusobacteria Fusobacterium ↑	0.033

(EMVI = Extra mural vascular invasion, LVI = Lymphovascular invasion).

**Figure 3:**
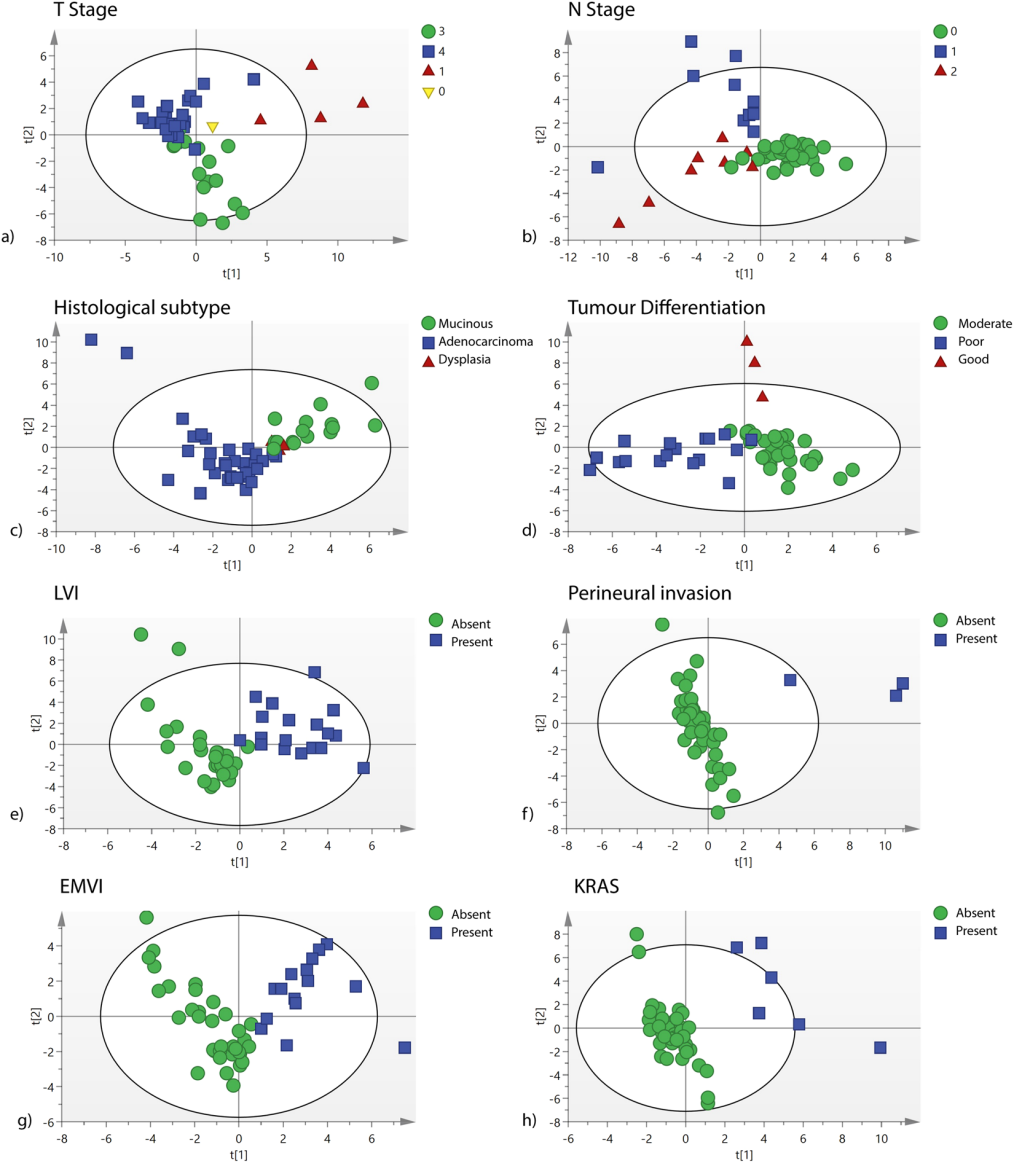
(**a**–**h**) Partial Least Squares-discriminant analysis scores plots of OTU data for important prognostic features identified by histopathological and molecular analysis. LVI = Lymphovascular invasion. EMVI = Extramural vascular invasion. PNVI = Perineural vascular invasion.

A multivariate analysis of the entire data set was then performed to determine if the data had clinical utility. Principle component analysis (PCA) identified three outliers (two from a patient with tubular villous adenoma and one from a patient with a T4 tumour) and these samples were removed, leaving 51 for further analysis (Supplementary data, Fig. 1). Supervised analysis was performed using Partial Least Squares discriminant analysis (PLS-DA) of mucosal ecology and models were built for all histological features of prognostic significance (Fig. 3). This analysis demonstrated that discrete clustering of samples was possible for all features analysed (Fig. 3a to h). Leave one sample out cross-validation revealed that the diagnostic accuracy was high for each feature (Supplementary data, Table 3). Reciever operating curves were then created (Supplementary data, Fig. 2) for EMVI (Area under curve (AUC) = 0.95), LVI (AUC 0.97), tumour differentiation (AUC 0.95) and KRAS mutation status (AUC 1.0). These data suggest that tumours of a poor prognostic phenotype maintain conserved components of the microbiome that correlate with the degree of tumour invasiveness and histological biomarkers of relevance to clinical outcomes.

### 1NMR and metabolic network analysis

**Figure 4:**
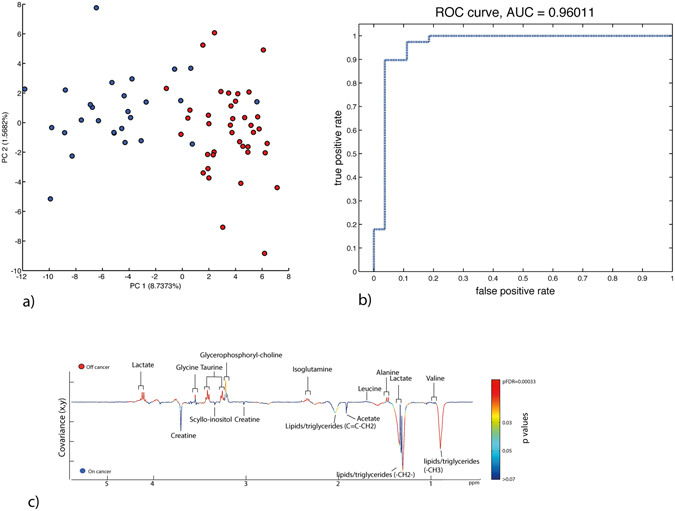
High Resolution – Magic Angle Spinning (HR-MAS) Nuclear Magnetic Resonance (NMR) analysis of mucosal metabolism using orthogonal partial least squares discriminant analysis. (**a**) Cross validated scores plot using Maximum Margin Criteria, and a leave one patient out validation. (**b**) The corresponding ROC curve demonstrating the diagnostic accuracy of the model (AUC 0.96). (**c**) Pseudo-loadings ANOVA plot of the MMC cross validated model, demonstrating co-variance of metabolites between on and off cancer sampling points. The colour code provides a visual description of FDR p values for each signal. Peaks point in the direction of the tissue state with which they are positively associated (up or down) and signals red in colour have greater statistical significance.

MAS-NMR analysis of tumour and healthy mucosa was performed as an untargeted analysis of mucosal metabolism during cancer progression. The summary data are reported in Fig. 4 and in Table 3.

**Table 3:** Summary of significant metabolites, their observed trends in terms of abundance (↑, ↓, ↔) with corresponding p-values, their chemical formulae and chemical shift assignments.

		Chemical structure	ppm	Expression in cancer	p
1	Acetate	-CH3	1.92(s)	↓	0.4
2	Alanine	-CH3	1.47 (d)	↑	0.0002
3	Creatine	-CH3, -CH2	3.02 (s), 3.93(s)	→	0.89
4	Formate	-CH2	8.45 (s)	↑	0.002
5	Glycerophosphorylcholine	N(CH3)3	3.22	↑	0.016
6	Glycine	CH2	3.56	↑	0.00005
7	Iso-butyrate	-CH3	1.0 (d)	↓	0.41
8	Isoglutamine	β-CH2	2.34	↑	0.001
9	Lactate	-CH3, -CH-	1.33 (d), 4.15 (q)	↑	7.69 × 10–9
10	Leucine	β-CH2 γ -CH	1.72	→	0.19
11	Lipid/Triglyceride	C = C-CH2	2.02	↓	0.02
12	Lipid/Triglyceride	1(CH2)n	1.29	↓	0.002
13	Lipid/Triglyceride	-CH3	0.9	↓	0.001
14	Phosphocholine	N(CH3)3, O-CH2	3.22(s), 4.19(t)	↑	0.007
15	Scylloinositol	-O-H	3.34	↑	0.67
16	Taurine	-CH2-NH, CH2SO3	3.26, 3.42	↑	2.01 × 10–9
17	Valine	-CH3	1.02	→	0.49

**Figure 5:**
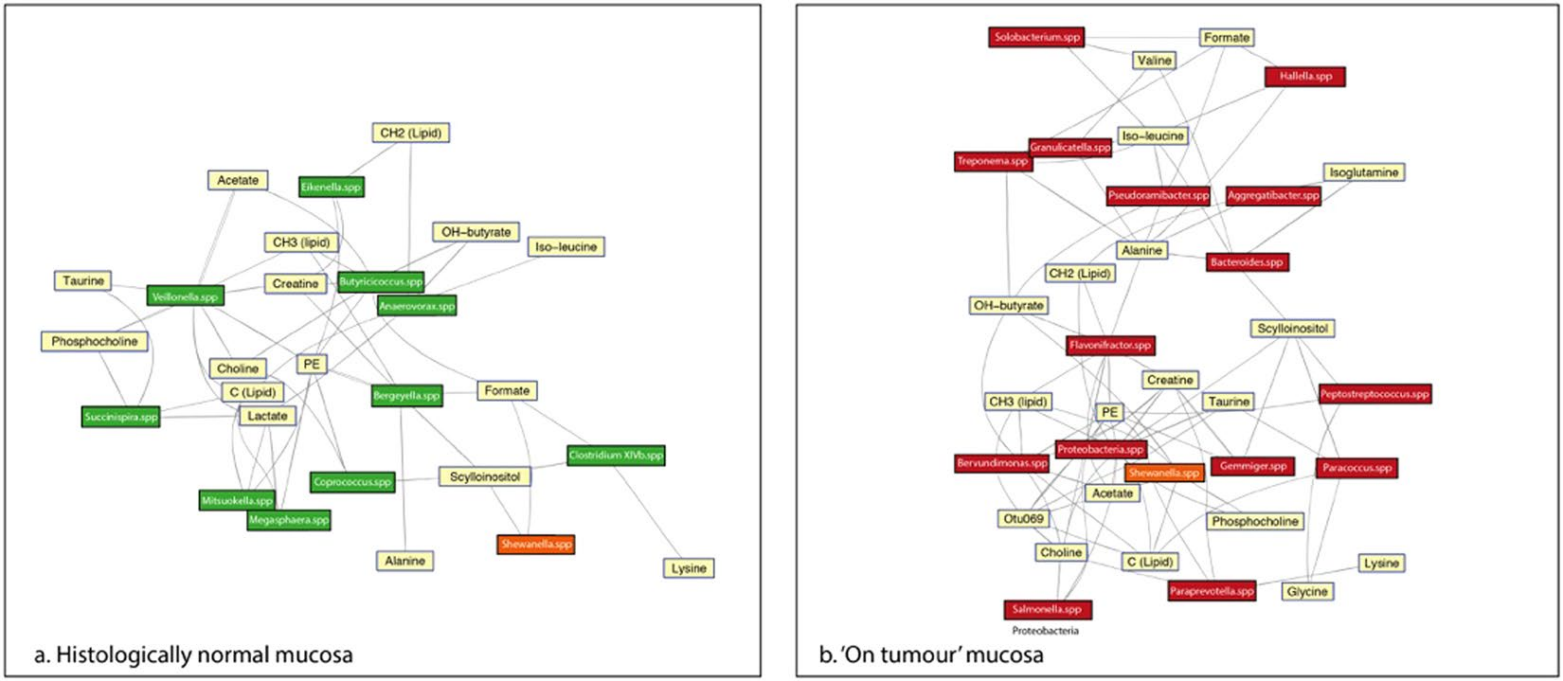
Metabolic network map of mucosal metabolites statistically over and under-represented in colon cancer and normal associated mucosa (NAM) as detected by ^1^H NMR-MAS regressed against the 16s rDNA OTU data. The spp. in green are microbiota over-represented in the NAM and those in red are over-represented in cancer. *Shewanella* spp. was the only bacteria class represented in both models.

As demonstrated in a previous study^21^, colonic mucosal metabolites are highly diagnostic of cancer (Fig. 4a and b). In contrast to the microbiome data presented here, clear metabolic separation was demonstrated between tumour and off tumour mucosal samples. Lipids and triglycerides were statistically important metabolic descriptors (Fig. 4c) for defining this variation and specifically they were positively correlated with on tumour metabolism. Molecules that showed significant statistical correlation with tumour or normal mucosal tissue status are shown in Table 3. These molecules were then regressed against the OTU data set to determine mucosal microbiome-metabonome functional associations that may be important in the maintenance of the cancer mucosal metabolic environment (Fig. 5). This analysis demonstrated that the network differs between on tumour and off tumour (normal healthy mucosa) sites. Only *Shewanella* spp. demonstrated commonality between these two tissue classes. OTUs associated with poor histological prognostic biomarkers did not demonstrate any statistical correlation, with the exception of Proteobacteria. Spp., which appeared to be linked to lipid, choline, taurine, acetate, and creatine metabolite expression. The tumour network did demonstrate greater network connectivity than the off tumour network, suggesting that the bacteria residing ‘on tumour’ have a greater catalogue of metabolic functions.

A secondary analysis was then performed to determine if these associations were consistent according to tumour stage. This analysis was limited by the small sample size for each stage, so no statistically significant interpretation was achieved (Supplementary data, Fig. 3). Correlations for Stage 0/1, 3 and 4 based on a p-value threshold of 0.05 demonstrated that only *Comamonadaceae acidovrax* spp. was consistently correlated with lipid metabolism across all stages of cancers. This Gram negative, aerobic genus is enriched in the mucosal biopsies of patients with inflammatory bowel disease, but its role in colon cancer is not established^22^.

## Discussion

Several species of pathobionts have now been implicated in the aetiology of colon cancer^23, 24^. In particular, the oral Gram negative bacterium *Fusobacterium nucleatum* has been strongly associated with adenomas of the colon and rectum^12, 15, 16, 25^. Moreover, there is evidence that the presence of *Fusobacterium* may be of prognostic importance as it is associated with CIMP positivity, TP53 wild-type, hMLH1 methylation positivity, Microsatellite Instability (MSI), and CHD7/8 mutation positivity^26^. However, many of the conclusions of such studies have been limited by methodological issues such as heterogeneity in sampling protocols and relatively small numbers of patients. Perhaps most importantly, there is often a lack of adequate clinical phenotyping data, and a false assumption that all colon cancers are homogeneous. The current study has therefore attempted to address some of the major confounders in translational microbiome research, including our uniform sampling of tumours without the use of bowel preparation. In preference to the faecal microbiota, we have targeted the CRC mucosal microbiome to provide a focussed analysis of histological prognostic factors that inform current clinical practice as part of a prospective clinical study.

Under these conditions, we have replicated key findings from other studies, including that the bacterium *F. nucelatum* is over represented on colorectal tumours. However, a major conclusion of our work is that mucosal populations are dynamic with advancing cancer stage, and that the CRC microbiome story is therefore more complex than the over representation of a small number of organisms. There was no significant variation in the microbiome ecology of normal associated mucosa over relatively short distances from the primary tumour site. This finding is in keeping with recent data from a larger cohort of patients^27^ and suggests that dysbiosis in colorectal cancer may pervade the whole colon. Inter-individual variation is marked and based on this analysis, true ‘healthy’ mucosa should be biopsied from consistent geographically discrete regions from non CRC-bearing individuals to provide a more representative ‘control’ sample of normal colonic ecology.

The data pesented here have also demonstrated more evidence in concordance with the colorectal cancer driver passenger theory^7^. Cancers of a poor prognostic phenotype were more likely to have over-representation of *Lachnospiracea intertie sedis, Streptococcus*, *Prevotella* and paraprevotella, (Table 2, Fig. 2). Individuals who demonstrated ecological similiarity between on and off cancer sites were more likely to have earlier cancers or tumours with better prognostic features.

Despite the dominance of inter-individual variation of microbiomes, using multivariate statistical models it was possible to define clear associations between the 16 s rRNA gene OTU data and tumour stage (T and N stage) (Fig. 3). Moreover, it was possible to define associations with established histopathological biomarkers of poor prognosis. Univariate interpretation of the data sets only demonstrated a small number of statistically meaningful associations with bacterial classes, with over-representation of *Bacteroides* spp., *Aggregatibacter* spp., *Streptoccocus* spp., *Clostridium* XI, *Solobacterium* spp. and *F. nucleatum* spp. associated with poor prognostic features. These associations do not imply a causative link, but in the context of the cancer stage data, they suggest that the advancing CRC microbiome contains over-represented ‘passenger’ elements (described previously) which may have clinical relevance and utility.

The structure of the CRC microbiome is increasingly well-defined, but its function in this context is less well understood. It is highly likely that the combined metabolic function of the mucosal network of bacteria plays a critical role in defining its impact on cancer initiation and progression^3^. Indeed a recent study of human fecal samples demonstrated strong microbe-metabolite correlations in CRC patients^28^. However, this metabolic function requires greater elucidation. We have previously demonstrated that the mucosal metabonome has a high diagnostic sensitivity and specificity between ‘on’ and ‘off’ cancer samples^20, 21^, which is defined by disruption to lipid metabolism. The diagnostic accuracy of MAS-NMR spectroscopy for CRC was replicated in the current analysis (Fig. 4), and subtle metabolic changes driven by microbial co-metabolism are integral to the model’s strength. We were able to correlate this metabolic data with 16 s rRNA gene sequencing to assess if the microbiota influences metabolic pathways on and off cancer. We have shown that the metabonome-microbiome metabolic network varies considerably between on and off tumour sites. This is only a statistical association, but it suggests that the mucosal microbiome has an important part to play in the maintenance of the cancer metabolic environment. Interestingly, the microbiota associated with histological features of poor prognosis were not associated with the metabolites which featured in the network analysis. This finding may imply that such classes of bacteria exert pathological influence via other molecular pathways, or that these are ‘passenger’ microbiota that reside on the mucosa of colorectal cancers and have a metabolic function that does not relate to tumour progression. The proteobacteria OTUs represented a statistically significant node on the network map, correlating with lipid, phosphocholine and taurine metabolism, which are strong metabolic biomarkers for cancer. Only the *Shewanella* spp demonstrated commonality between on and off tumour positions. These are marine bacteria, which have been shown to possess enzyme functions reminiscent of eukaryotic pepsin homologues^29^.

Abnormal choline metabolism is emerging as a metabolic hallmark associated with oncogenesis and tumour progression. We have previously identified choline as a biomarker of colon cancer risk in susceptible individuals associated with altered microbiome metabolism^3^. Microbiome modulated metabolism of choline is also closely associated with cardiovascular disease^30^. Phosphocholine is both a precursor and a breakdown product of phosphatidylcholine, which, together with other phospholipids such as phosphatidylethanolamine and neutral lipids, forms the characteristic bilayer structure of cellular membranes and regulates membrane integrity. It is not clear whether altered choline metabolism, and secondary choline metabolites, influence microbiome abundance in CRC, or vice versa. The small number of patients with early cancer in this study means that subgroup analysis was not possible. Nevertheless, this work provides further evidence that microbiome modulation of the choline metabolic pathway is an important influence on the cancer metabolic niche.

There are some obvious limitations with this work. Firstly, the sample size is small, particularly for patients with specific molecular features such as KRAS mutation. 16 S rRNA gene analysis does not permit strain level assignment of bacteria and as a result, it is not possible to investigate the specific metabolic functions of target strains. In this study we applied a non-targeted ^1^H HR-MAS NMR approach to the metabolic analysis, so we have not been able to report on certain established metabolites known to have important pro or anti-neoplastic effects (e.g. bile acids and short chain fatty acids). We also acknowledge that linking metabolic data such as these to taxanomic data is statistically challenging using clinical sample sets. But despite these limitations, the present work has identified novel bacterial classes associated with adverse histopathological features in CRC and it has provided more data to support the ‘driver - passenger model’ as an important mechanism in the aetiology and progression of colon cancer. Moreover, ours is the first study to use 1 H MAS-NMR data linked to a robustly sampled 16 S rRNA data set from well phenotyped patients and it provides a basis for the metabolic function of commensal bacteria at the level of the mucosa in cancer.

In conclusion, the cancer mucosal microbiome is individualized, and evolves with cancer stage to meet the demands of cancer metabolism. In addition to normal associated mucosa from CRC patients, which may not be representative of a healthy gut microbiome, future studies should also use control samples from non CRC-bearing individuals. It remains to be proven that ‘driver’ species of the mucosal microbiota modulate cancer initiation, but this study suggests that ‘passenger’ bacteria in the evolving CRC microbiota may play a role in the maintenance of tumoral metabolic homeostasis and could serve as useful clinical biomarkers.

## Methods

### Patient recruitment and sampling

Between November 2011 and September 2012 tissue specimens and related clinico-pathological data were collected with informed written consent from 18 patients undergoing planned surgical resection for right sided colorectal cancer, at a single cancer referral center (St Mary’s Hospital (London, UK)). Inclusion criteria were: patients with histologically confirmed invasive malignancy or high-grade dysplasia of the colon, having either open or laparoscopic surgery without the use of bowel preparation. Exclusion criteria were: patients undergoing emergency surgery, patients treated with neoadjuvant chemotherapy and/or radiotherapy, patients who had been on antibiotics or probiotic therapies within the previous six weeks and patients with rectal cancers (defined here as tumours lying within 15 cm of the anal verge). Patients with a history of Familial Adenomatous Polyposis (FAP) were also excluded as were patients with inflammatory bowel disease or those who had undergone previous colorectal surgery. At induction, patients had an intravenous dose of cefuroxime and metronidazole as per standard local hospital protocol. At surgery, fresh tissue samples were harvested from the tumour centre and at 5 cm and 10 cm away from the tumour. Tissue harvesting was performed in the pathology department by a single gastrointestinal histopathologist (RDG) and acquired samples were immediately transferred to a freezer at −80 °C.

### DNA Extraction and profiling of 16 S rRNA genes

Total DNA was extracted from biopsies using MO BIO’s powersoil DNA isolation kit. The V1-V3 regions of the 16 S rRNA genes were amplified (28 F 5’-GAGTTTGATCNTGGCTCAG and 519 R 5’-GTNTTACNGCGGCKGCTG) and sequenced on a Roche 454 platform by Research and Testing Laboratory (Austin, Texas, USA). The sequences were processed using Mothur to remove low quality sequences and chimeras^31, 32^. All samples were normalized to the lowest number of reads using the subsample command in Mothur and alpha and beta diversity indices were calculated. All other statistical analysis and multi-variate analysis of the 16 S rRNA profiles was performed in R.

### qPCR to enumerate Fusobacterium nucleatum in extracted DNA

Biopsy colonisation by *Fusobacterium nucleatum* was assessed by qPCR of the 16 S rRNA genes in the DNA extracts normalised to sample total generic pan bacterial 16 S rRNA copies, expressed as the difference in respective qPCR threshold emergence cycle time (Ct) of generic and specific *Fusobacterium nucleatum*. Ct is inversely proportional to copy number so the smaller the difference the greater the relative quantity of specific *Fusobacterium nucleatum* DNA and hence the degree of biopsy colonisation.

*Fusobacterium nucleatum* primers were 527 F GGATTTATTGGGCGTAAAGC and 689 R GGCATTCCTACAAATATCTACGAA^16^. Pan bacterial 16 S qPCR primers were 343 F TCCTACGGGAGGCAGCAGT and 809 R GGACTACCAGGGTATCTAATCCTGTT. The qPCR used Maxima Mastermix SYBR green (Thermo-Fisher) with lots pre-screened for minimal endogenous bacterial 16 S DNA background sufficient to permit 30 PCR cycles. Cycling details: 95 °C 10 minutes, then 30 cycles of 95 °C 15 seconds and 60 °C 60 seconds. Melt curve analysis was used to determine amplicon fidelity.

### MAS NMR sample preparation

Tissue samples were kept on ice at all times during the preparation process to minimize metabolite degradation. Where tissue volume permitted, 2 replicates were prepared for High Resolution – Magic Angle Spinning (HR-MAS) Nuclear Magnetic Resonance (NMR) analysis from each original tissue sample, to compensate for anticipated tissue heterogeneity. Sampling was performed using a disposable punch biopsy device, after which 12–15 mg of tissue was packed into disposable 30 µL Teflon NMR inserts. Deuterated water (D_2_O) was next added to the insert to complete required volume and homogenize insert contents. Inserts were introduced into zirconium oxide rotors for analysis. The spectroscopic profiling approach employed in the present study has been previously described by our group^21^. In summary, 1-dimensional ^1^H Carr-Purcell-Meiboom-Gill (CPMG) spectra were acquired using a Bruker Avance III 400-MHz spectrometer equipped with magic angle spinning probe (Bruker BioSpin GmbH, Rheinstetten, Germany). A water suppression pulse was applied during experimentation to minimize the water signal. Acquired spectra were processed using Bruker software packages (TopSpin v.2.2 and Amix v.3.9.9), and the methyl signal of alanine was used for spectral calibration (1.47 ppm). Spectral metabolite identification and chemical assignment were performed on the basis of the literature^21, 33, 34^ and using statistical total correlation spectroscopy approaches for data-driven structural assignment^35, 36^.

### Statistical analysis

Phased and baseline corrected CPMG spectra were converted into statistical matrices using in-house tools developed in MATLAB (v.7.12.0.635; The MathWorks, Inc., Natick, MA, USA). The matrix contained information from the region −1 to 10 ppm, and the resolution used was 0.00055 ppm^21^. Spectral peaks corresponding to water (4.50–5.19 ppm), ethanol (1.10–1.20, 3.60–3.90 ppm), and polyethylene glycol (3.70–3.75 ppm) signals were excluded from the analysis because these chemical regions do not provide biologically relevant information. Spectral pre-processing involved probabilistic quotient normalization and unit variance scaling. Processed data were subjected to both univariate and multivariate analyses. For univariate analysis, we used analysis of variance to identify the statistical significance (reported as *P* values) of the discriminatory capacity of individual metabolic features. Ecological analysis was performed using the Tau (for similarity) and Shannon indices (for community diversity). Data were normalized to the on tumour value for each individual. Community clustering was assessed using the Bray-Curtis distances of the genus-level OTU reads, using the Ward linkage algorithm.

For multivariate analysis, we used unsupervised principal component analysis (PCA) and supervised orthogonal partial least squares–discriminant analysis (OPLS-DA) using in-house developed scripts operating in a MATLAB environment. For each generated OPLS model cross-validated *Q* ^2^ values were obtained before model robustness was further assessed by calculating the area under the curve (AUC) from corresponding receiver operating characteristic (ROC) curves^37^. Where OPLS score scatter plots revealed separation between classes, loadings plots were generated and assessed to identify the metabolites most responsible for discrimination. Additionally, correlation-driven network analysis was used to construct dependencies between metabolites in tumor and healthy mucosal tissues. The correlation coefficients between metabolites were calculated *via* non-parametric Spearman metrics. The spring embedding algorithm was used to calculate the optimum layouts of metabolite correlation networks^38^.

### Ethics approval and consent to participate:

This study was granted full ethical approval by the institutionalreview board at Imperial College Healthcare NHS Trust (REC reference number 07/H0712/112). All experiments were carried out in accordance with relevant guidelines and regulations.

### Availability of data and material:

The 16 S rRNA gene sequences supporting the results of this article is available at the EBI’s ENA short read archive under number PRJEB13249.

## Electronic supplementary material

Supplementary Information
