# Supplementary material for: A prospective analysis of mucosal microbiome-metabonome interactions in colorectal cancer using a combined MAS 1HNMR and metataxonomic strategy
Source: Sci Rep. 2017 Aug 21;7:8979. doi: 10.1038/s41598-017-08150-3 (PMC5566496; doi:10.1038/s41598-017-08150-3)
Supplement: Supplementary file 1 — Supplementary Information [file 41598_2017_8150_MOESM1_ESM.pdf]

Supplementary data

A prospective analysis of mucosal microbiome-metabonome interactions in colorectal cancer using a combined MAS 1HNMR and metataxonomic strategy.

James Kinross[1], Reza Mirnezami[1], James Alexander[1], Richard Brown[5], Alasdair Scott[1], Dieter Galea[2], Kirill Veselkov[2], Rob Goldin[3], Ara Darzi[1], Jeremy Nicholson,[2] Julian R. Marchesi*[4, 5].

1. Biosurgery and Surgical Technology, Department of Surgery and Cancer, Imperial College London, UK.

2. Computational and Systems Medicine, Faculty of Medicine, Department of Surgery and Cancer, Imperial College London, UK.

3. Centre for Pathology, Faculty of Medicine, Imperial College London, UK.

4. Division of Digestive Diseases, Faculty of Medicine, Department of Surgery and Cancer, Imperial College London, UK

5. School of Biosciences, Cardiff University, Cardiff, UK

	Cluster 1	Cluster 2	Cluster 3	Significance (p=)
Location				0.842
10cm	10	5	3	
5cm	10	5	3	
Tumour center	13	3	2	
N stage				0.097
0	24	6	3	
1	7	3	2	
2	2	4	3	
T stage				0.04*
0	6	3	0	
1	3	0	0	
2	0	3	0	
3	12	3	0	
4	12	4	8	
Histo subtype				0.05*
Adeno	12	10	8	
Mucinous	12	3	0	
Dysplasia	9	0	0	
Differentiation				0.011*
None	3	0	0	
Moderate	17	13	3	
Poor	13	0	5	
LVI				0.072
None	19	9	8	
Present	14	4	0	
Perineural invasion				
None	30	13	8	0.364
Present	3	0	0	
KRAS				
Wild type	29	11	8	0.529
Mutation	4	2	0	
EMVI				
None	24	9	6	0.982
Present	9	4	2	

Table 1. Oncological features of three mucosal ‘enterotypes’. T stage, histological subtype and tumour differentiation were statistically significant for differentiating between classes. Cluster 1 and 2 contained patients with dysplastic lesions and earlier cancers, and class 2 only contained moderately differentiated tumours. The third cluster of 8 patients was only made up T4 adenocarcinomas, with poor tumour differentiation and a trend towards nodal metastases. LVI = Lymphovascular invastion. EMVI = Extramural vascular invasion.

	Clustered	Not Clustered	Significance (p=)
Location			1.00
10cm	6	12	
5cm	6	12	
Tumour center	6	12	
N stage			0.017*
0	15	18	
1	0	12	
2	3	6	
T stage			0.073
0	3	6	
1	0	3	
2	3	0	
3	6	9	
4	6	18	
Histo subtype			0.054
Adeno	9	21	
Mucinous	3	12	
Dysplasia	6	3	
Differentiation			0.038*
None	3	0	
Moderate	6	24	
Poor	9	12	
LVI			0.066
None	15	21	
Present	3	15	
Perineural invasion			0.208
None	18	33	
Present	0	3	
KRAS			0.358
Wild type	15	33	
Mutation	3	3	
EMVI			0.197
None	15	24	
Present	3	12	

Table 2: Oncological prognostic features of patients whose on and off tumour samples clustered together. Nodal involvement and tumour differentiation were statistically significant descriptors of differences between the two cohorts, with non-significant trends noted for T stage, histological subtype and LVI.

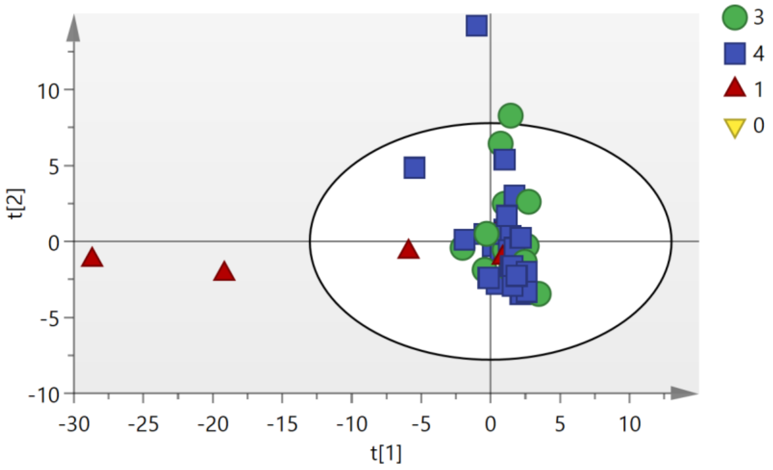

Figure 1. PCA plot of all samples according to tumour stage (R2X = 0.278, Q2X = 0.07). The three outliers were removed from the remaining analysis, leaving 51 samples in total.

	Samples	PCs	R2Y	Q2Y	Diagnostic accuracy %
T stage	51	2	0.46	0.18	84.3
N stage	51	2	0.58	0.339	88.2
Histo subtype	51	2	0.46	0.274	90.3
Differentiation	51	2	0.65	0.273	90.25
LVI	51	2	0.79	0.429	98.04
Perineural invasion	51	2	0.89	0.659	100
EMVI	51	2	0.802	0.293	94.12
KRAS	51	2	0.893	0.511	100

Table 3. Summary data for the Partial Least Squares Discriminant multivariate models. PC= Principle component. Overall diagnostic accuracy of the model is demonstrated based on the creation of a confusion matrix. LVI = Lymphovascular invasion. EMVI = Extramural vascular invasion.

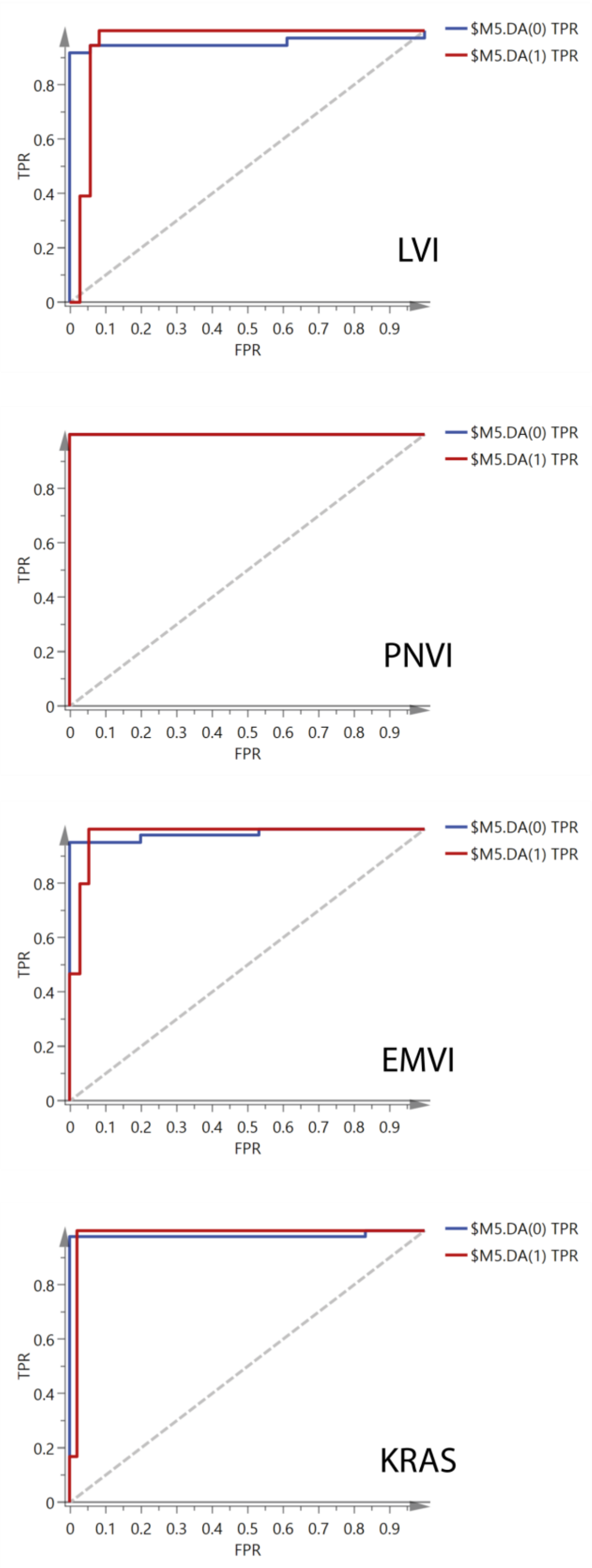

Figure 2: Summary receiver operating curves (ROC) curves built for each prognostic model with two diagnostic variables. LVI = Lymphovascular invasion. EMVI = Extramural vascular invasion. PNVI = Perineural vascular invasion.

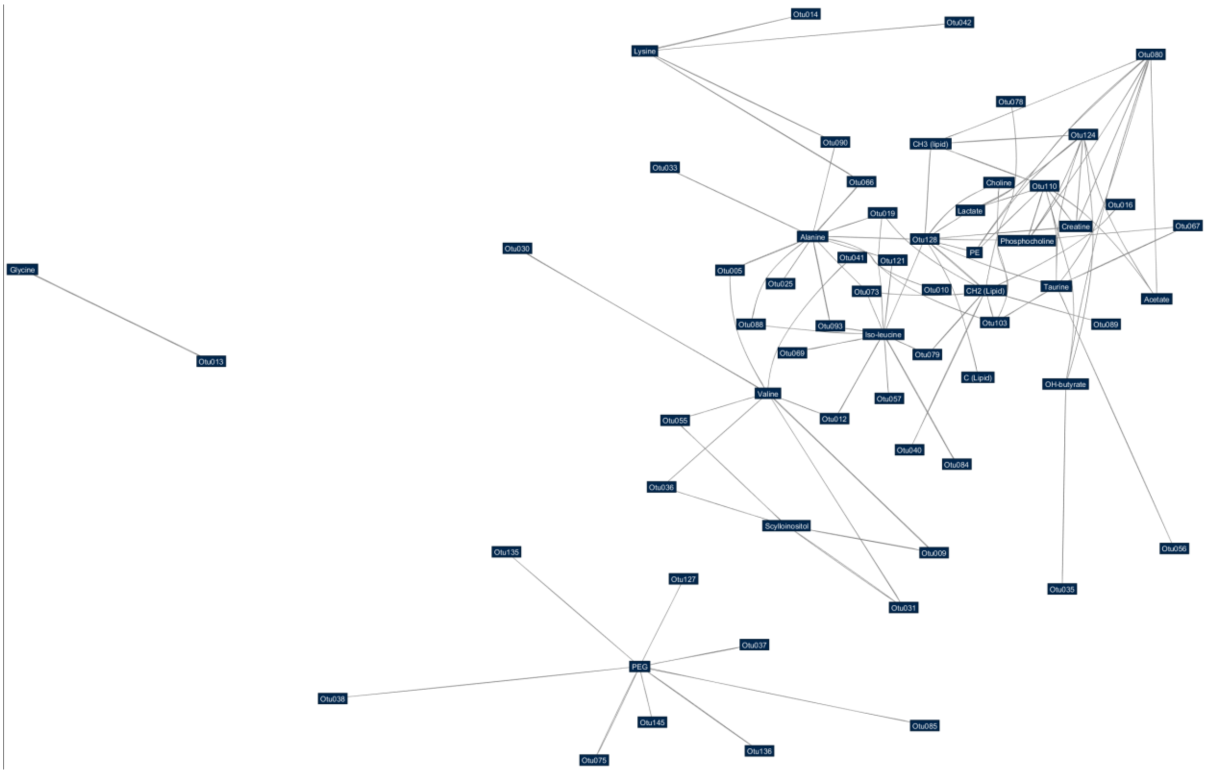

Otu014
Otu042
Lysine
Otu080
Otu078
Otu090
CH3 (lipid)
Otu124
Otu033
Otu066
Choline
Otu110
Lactate
Otu016
Otu019
Creatine
Otu067
Otu030
Alanine
Otu128
Phosphocholine
PE
Glycine
Otu005
Otu041
Otu121
Otu025
Otu073
Otu010
CH2 (Lipid)
Taurine
Acetate
Otu088
Otu093
Iso-leucine
Otu103
Otu089
Otu013
Otu069
Otu079
C (Lipid)
OH-butyrate
Valine
Otu057
Otu055
Otu012
Otu040
Otu084
Otu036
Scylloinositol
Otu009
Otu056
Otu135
Otu127
Otu035
Otu031
Otu037
PEG
Otu038
Otu145
Otu085
Otu136
Otu075

a)

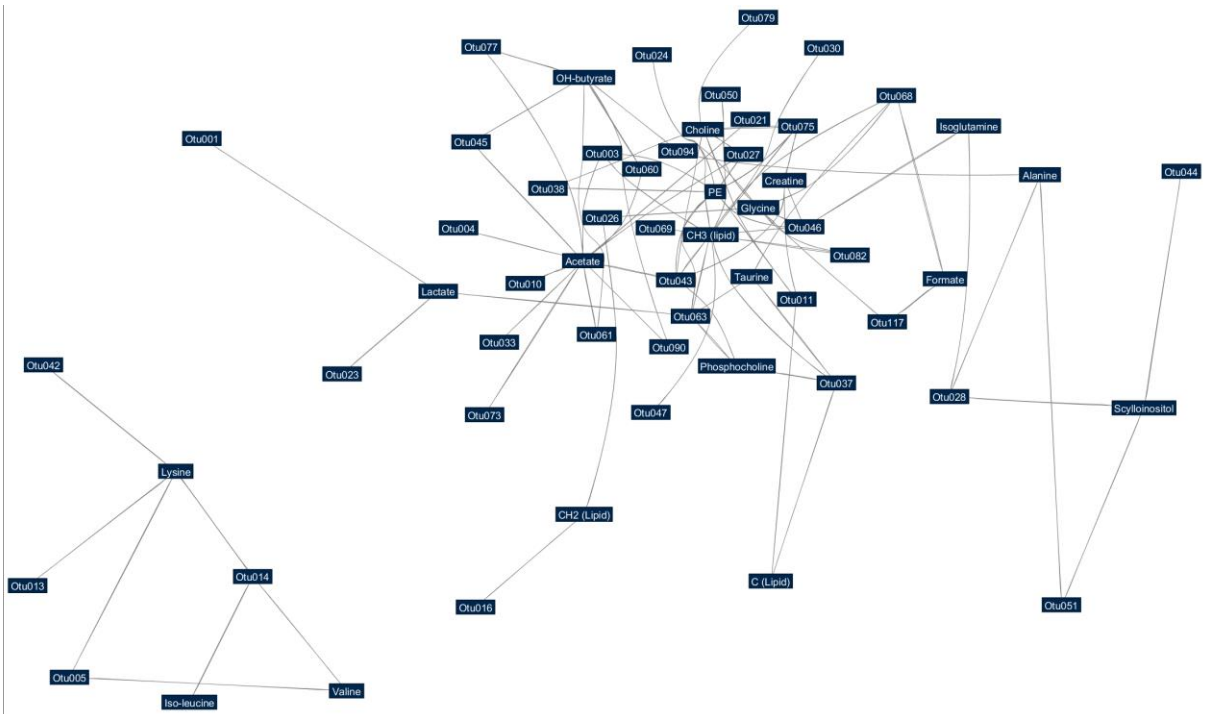

Otu079
Otu077
Otu024
Otu030
OH-butyrate
Otu050
Otu068
Otu021
Choline
Otu075
Isoglutamine
Otu001
Otu045
Otu003
Otu094
Otu027
Otu060
Creatine
Alanine
Otu044
Otu038
PE
Glycine
Otu026
Otu004
Otu069
CH3 (lipid)
Otu046
Otu082
Acetate
Otu010
Otu043
Taurine
Formate
Lactate
Otu011
Otu063
Otu117
Otu061
Otu033
Otu090
Otu042
Otu023
Phosphocholine
Otu037
Otu073
Otu047
Otu028
Scylloinositol
Lysine
CH2 (Lipid)
Otu013
Otu014
C (Lipid)
Otu016
Otu051
Otu005
Valine
Iso-leucine

b)

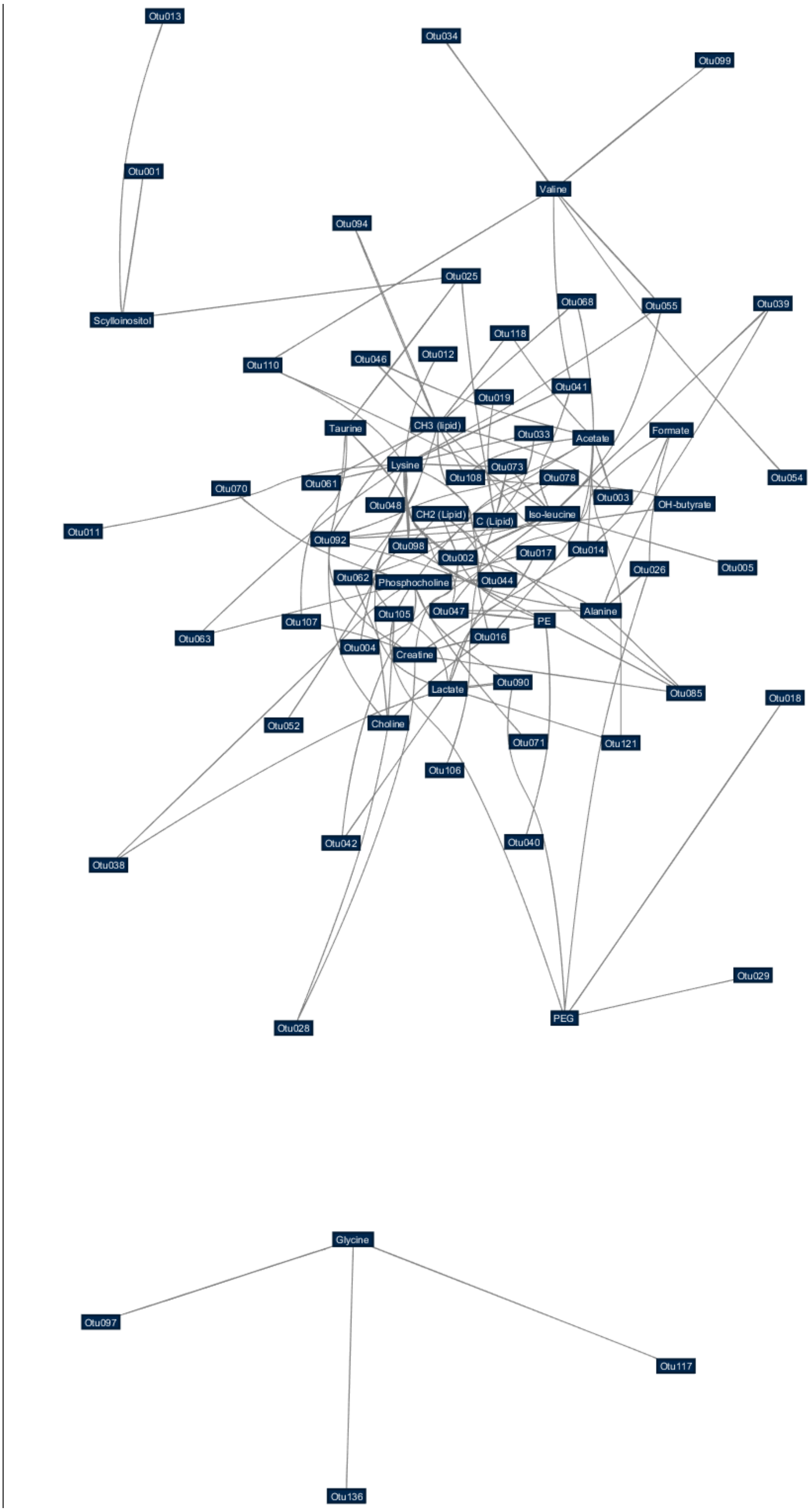

c)

Figure 3. 1H MAS-NMR / OTU Correlation networks for a) Stage 0/1 b) stage 3 and c) stage 4 cancers, visualized separately according to a p-value threshold of 0.05.
